# Paper-based genetic assays with bioconjugated gold nanorods and an automated readout pipeline

**DOI:** 10.1038/s41598-022-10227-7

**Published:** 2022-04-13

**Authors:** Claudia Borri, Sonia Centi, Sofia Chioccioli, Patrizia Bogani, Filippo Micheletti, Marco Gai, Paolo Grandi, Serena Laschi, Francesco Tona, Andrea Barucci, Nicola Zoppetti, Roberto Pini, Fulvio Ratto

**Affiliations:** 1 Istituto di Fisica Applicata “Nello Carrara”, Consiglio Nazionale delle Ricerche, 50019 Sesto Fiorentino, FI Italy; 2 Dipartimento di Biologia, Università degli Studi di Firenze, 50019 Sesto Fiorentino, FI Italy; 3 Laboratori Victoria S.R.L, 51100 Pistoia, Italy; 4 Ecobioservices & Researches S.R.L, 50019 Sesto Fiorentino, FI Italy

**Keywords:** Biotechnology, Computational biology and bioinformatics, Health care, Medical research, Materials science, Optics and photonics

## Abstract

Paper-based biosensors featuring immunoconjugated gold nanoparticles have gained extraordinary momentum in recent times as the platform of choice in key cases of field applications, including the so-called rapid antigen tests for SARS-CoV-2. Here, we propose a revision of this format, one that may leverage on the most recent advances in materials science and data processing. In particular, we target an amplifiable DNA rather than a protein analyte, and we replace gold nanospheres with anisotropic nanorods, which are intrinsically brighter by a factor of ~ 10, and multiplexable. By comparison with a gold-standard method for dot-blot readout with digoxigenin, we show that gold nanorods entail much faster and easier processing, at the cost of a higher limit of detection (from below 1 to 10 ppm in the case of plasmid DNA containing a target transgene, in our current setup). In addition, we test a complete workflow to acquire and process photographs of dot-blot membranes with custom-made hardware and regression tools, as a strategy to gain more analytical sensitivity and potential for quantification. A leave-one-out approach for training and validation with as few as 36 sample instances already improves the limit of detection reached by the naked eye by a factor around 2. Taken together, we conjecture that the synergistic combination of new materials and innovative tools for data processing may bring the analytical sensitivity of paper-based biosensors to approach the level of lab-grade molecular tests.

## Introduction

In the last 20 years, plasmonic nanoparticles have sparked more and more interest for their chemical and optical versatility, biocompatibility, stability and efficiency as optical labels or contrast agents for biosensing, biomedical imaging, Raman or luminescence spectroscopy, the controlled release and delivery of drugs, the optical hyperthermia of cancer and many other applications^1–5^.

In particular, thanks to their photophysical properties and ease of chemical synthesis, gold nanoparticles (AuNPs) stand out as an excellent choice to develop a plethora of colorimetric biosensors. The optical features of colloidal suspensions of AuNPs depends on numerous parameters, i.e. their size, shape, state of aggregation and environment^3,6,7^. It is relatively simple to modify their surface with functional biomolecules like proteins^8,9^, such as antibodies^10,11^ and enzymes^12,13^, aptamers^14,15^, other oligonucleotides^16,17^, or polysaccharides^18–20^. Relevant methods include protocols for both passive absorption (i.e. van der Waals, ionic or hydrophobic interactions) and the formation of covalent bonds (e.g. amidation and thiolation) or their combination. Their refinement has reached the level that it has become possible to tweak parameters like the surface orientation of antibodies^9,21^.

The bright colors of AuNPs originate from localized surface plasmon resonance (LSPR) oscillations, and so exhibit sensitivity to subtle variations of their near-field landscape. By assessing their accumulation or spectral shift, it is indeed possible to monitor specific biomolecular interactions in suspension or on paper-based supports^22–26^. The LSPR phenomenon has been exploited in e.g. colloidal nanoparticle-based ELISA sensors^27^, colorimetric and multicolor immunoassays^25,26,28,29^, standard and advanced lateral flow assays^26,30,31^, and many more inventive methods. For instance, the aggregation of AuNPs has been used as a mechanism to modulate the enhancement of Raman signals^32^. The particular case of paper-based biosensors with immunoconjugated AuNPs has played an extraordinary role in the recent COVID-19 outbreak, as the technological platform implemented in the so-called rapid antigen-tests. However, in spite of their incredible success, these systems have also shown weaknesses: their analytical sensitivity amounts to several hundred units of Median Tissue Culture Infectious Dose (TCID_50_) per ml^33,34^, which is at least two to three orders of magnitude worse than that of Polymerase Chain Reaction (PCR)-based tests^35^, and their large-scale use is qualitative only.

Here, we describe a synergistic set of solutions that collectively aim at a more sensitive and quantitative use of paper-based biosensors with bioconjugated AuNPs. First, we focus on a genetic rather than a protein target. Second, we test the use of anisotropic AuNPs, such as gold nanorods (AuNRs). And third, we assess the feasibility of machine learning for a scenario of automatic readout. In order to keep control over all kinetic parameters, we implement a dot-blot setup as a convenient model of a paper-based assay.

The use of genetic targets in lateral flow assays provides the unique advantage of compatibility with PCR-based^36–38^ or isothermal^39–41^ protocols for amplification. The detection of genetic markers is a fundamental tool in various contexts, such as medical diagnostics, forensics, agriculture and environmental monitoring, e.g. to profile the microorganisms involved in pathogenic infections, to trace the diffusion of antibiotic resistance, or to identify genetically modified organisms of ecological concern. In this work, we chose the *rol*C gene as a well-known model of general interest. The *Agrobacterium rhizogenes rol* oncogenes are an ideal candidate for plant engineering^42^, and regulate their growth, cell differentiation and secondary metabolism in transformed cells from several botanical families^43^. It has also been suggested that the expression of these genes may have played a key role in plant evolution^44,45^ and in the establishment of new species^46^.

By coming in multiple and much brighter colors, AuNRs outperform their isotropic predecessors in several respects. The efficiency of optical absorbance of AuNRs is about 10 times larger than that of spherical AuNPs^6,47,48^, which translates into a better visibility and so an intrinsic potential for earlier detectability in colorimetric setups. In addition, the colors of AuNRs depend on their aspect ratio (AR = length/diameter), which is a programmable parameter in their synthesis, and so a degree of freedom available for multiplexing. The fabrication of AuNRs displaying AR greater than around 4 and resonating at deep-penetrating near-infrared frequencies is a recurrent solution in nanomedicine for applications like the optical hyperthermia and photoacoustic imaging of cancer^49–53^. However, when it comes to their functionalization with nucleotide probes, the switch from spherical AuNPs to AuNRs is nontrivial. In particular, differences arise because the electrokinetic potential of as-synthesized citrate-capped AuNPs is anionic, while it flips to strongly cationic in the case of standard cetrimonium-coated AuNRs. As a consequence, unmodified AuNRs are liable to undergo flocculation in the presence of DNA strands with their polyanionic backbone^54,55^. In this context, the modification of AuNRs with citrate^56,57^ has emerged as a smart shortcut for a smooth transfer of the protocols developed in many decades of work on spherical AuNPs, by emulating their surface-chemistry.

The application of artificial intelligence to the interpretation of dot-blot assays is a topical idea that may help fulfill their quantitative readout. The coloration pattern within each blot is often irregular and non-uniform, which complicates a traditional analysis of measurable signals like the intensity of diffuse optical reflectance^9^ or surface enhanced Raman scattering^58,59^, in the case of active tags. As a consequence, the typical use of these predictors is qualitative only or semi-quantitative, at best. Here, we assess the feasibility of a supervised machine learning approach to the regression of standardized photographs of dot-blots as a tool to go far beyond their qualitative readout. The application of artificial intelligence to the objective interpretation of biomedical images of all sorts, including e.g. micrographs, tomographs or endoscopies of cells^60^, tissues^61,62^ or patients^63,64^, is becoming a mainstream paradigm to identify hidden patterns and support clinical decisions at any level. In the context of paper-based assays, for instance, A. Carrio et al. developed a light box and a pipeline based on a multilayer perceptron artificial neural network for the classification of results from commercial lateral flow tests for the detection of drugs of abuse in the saliva^65^. Other authors used linear support vector machines to classify images of lateral flow strips^66,67^ or pH indicator papers^68^. However, the jump from classification between discrete bins to regression against a continuous scale remains a missing step in the path to analytical quantification.

Here, we propose a systems engineering perspective on the refinement of paper-based assays, where we pursue a holistic redesign of all components from materials to hardware and software, in order to leverage on new synergies between up-to-date developments made in complementary subfields of science and technology.

## Results and discussion

### Citrate-coated gold nanorods

The overall strategy that we chose to couple AuNRs to hybridization probes was to first modify their surface with citrate and then resort to the extensive literature on the conjugation of citrate-AuNPs to thiolated oligonucleotides^69,70^. However, our starting point was regular AuNRs coated with cetrimonium bromide (CTAB). By being a cationic surfactant, CTAB is widely used in extraction buffers to facilitate the purification of DNA from polysaccharides and pigments in plant tissues^71–73^. Thus, in order to minimize the nonspecific adsorption of the particles on non-targeted oligonucleotides, and to ensure their colloidal stability during their bioconjugation, cetrimonium-AuNRs were converted into citrate-AuNRs, which emulates the typical termination of spherical AuNPs. The substitution was done according to a variant of the protocol documented in detail by J. G. Mehtala et al.^56^, where the stabilizer is first replaced from CTAB to a weak adsorbate of poly(sodium 4-styrenesulfonate) (Na-PSS), and, from there, to a dense layer of Na_3_-citrate.

The electrokinetic potential and hydrodynamic size of citrate-AuNRs were analyzed by dynamic light scattering (DLS) and compared to their as-synthesized cetrimonium-stabilized counterpart. To perform these measurements, AuNRs were diluted in ultrapure water until a nominal concentration of 80 µM Au.

**Figure 1:**
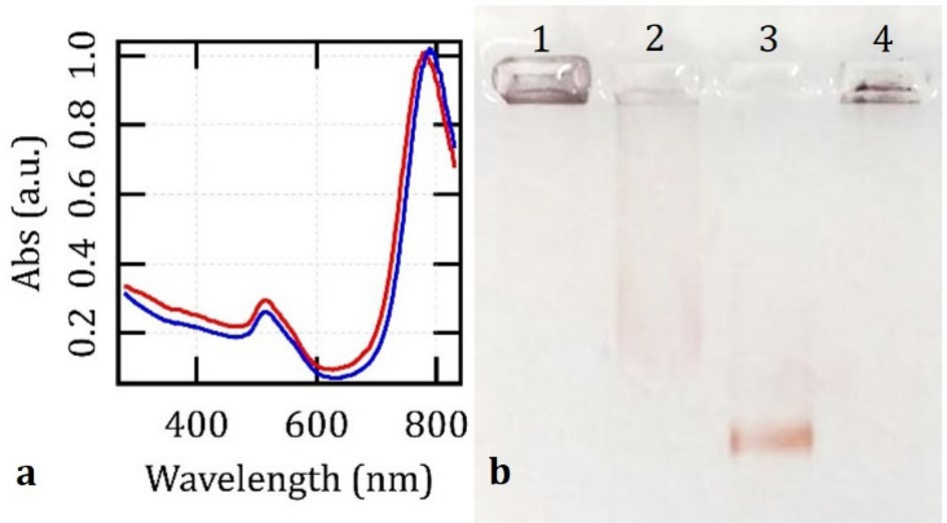
**(a)** Representative optical spectra of AuNRs at the nominal concentration of 160 µM Au in 0.5 mM CTAB and 0.005% Tween 20 (blue) or in 5 mM Na_3_-citrate (red). **(b)** Electrophoretic mobility of cetrimonium-AuNRs (1), citrate-AuNRs (2) and citrate-AuNRs functionalized with the 41-mer oligonucleotide (3) or the 328-mer single-stranded sequence (4) in 1% agarose gel under 100 V for 60 min in TBE buffer. The original picture of the agarose gel is reported in Fig. S4 (A).

The mean values of their ζ potential and average diameter respectively changed from (+ 30.9 ± 3.0) to (− 28.4 ± 2.1) mV, which is in nice agreement with the data reported in Ref.^56^, and from (100.2 ± 3.9) to (98.1 ± 3.4) nm. The optical spectra of the AuNRs displayed two characteristic peaks: a longitudinal band centered around 790 nm and a transversal one about 516 nm. As shown in Fig. 1a, the extinction spectra of the colloids coated with citrate or cetrimonium were almost indistinguishable. When subjected to a 1% agarose gel electrophoretic run, 3.6 mM Au citrate-AuNRs migrated towards the anode for a distance around 1.5 cm in 0.5 × Tris–Borate EDTA buffer (TBE, pH 8.3) at 100 V for 60 min (Fig. 1b, sample 2). On the other hand, cetrimonium-AuNRs failed to leave their well, probably due to the onset of flocculation under the ionic strength of the TBE buffer (sample 1). (Note that the pore size of a 1% agarose gel was estimated to range from 100 to 500 nm^74,75^). Taken together, these data demonstrate the successful replacement of CTAB with Na_3_-citrate, while preserving the colloidal stability and optical properties of the AuNRs. We found that these colloids remained stable and usable for several months when stored at shelf conditions.

### Bioconjugated AuNRs

**Figure 2:**
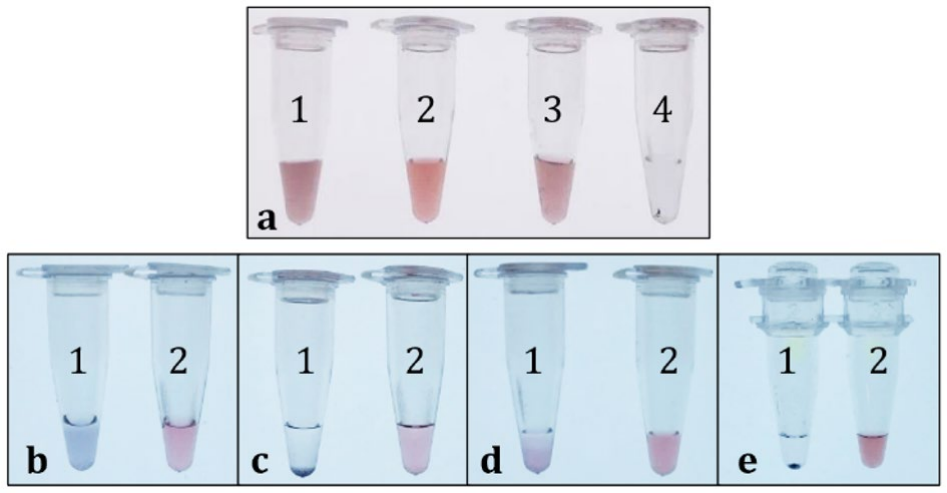
**(a)** Representative images of 0.8 mM Au citrate-AuNRs. (1) control particles, (2) AuNRs functionalized with the 41-mer oligonucleotide + NaCl, (3) AuNRs conjugated to the 328-mer single-stranded fragment + NaCl, (4) control particles + NaCl. **(b–e)** Control AuNRs (1) and AuNRs conjugated to the 41-mer oligonucleotide (2) re-suspended in DIG Easy Hyb buffer ((**b**) 5 min and (**c**) 24 h) or in DPBS ((**d**) 3 h) at a nominal concentration of 0.8 mM Au at RT, and in a typical PCR master mix at a rate of 1.6 mM Au after 25 thermal cycles (**e**).

In this work, we chose the *rol*C gene as a model of target analyte, and we tested two different nucleotide probes for bio-conjugation of the AuNRs. The first probe was a synthetic 21-mer forward primer designed for amplification of the *rol*C gene in end-point PCR, which was modified with a tail of 20 thymine bases and a terminal thiol at its 5′-end (see Table 1). The second probe was a 328-mer single-stranded sequence derived from the elongation of said primer in a dedicated PCR reaction. In both cases, the bioconjugation of the AuNRs was achieved by incubation with the thiolated sequences, in the presence of NaCl. A high ionic strength proved to be essential to mitigate the electrostatic repulsion between the citrate-AuNRs and the nucleotide probes, and to promote a proper functionalization. In order to avoid destabilizing the suspension, salt was added in small aliquots at regular intervals over 2 days. While the bioconjugation progressed, the color of the AuNRs became even more crimson and brilliant (Fig. 2a, sample 2) than the control colloids (Fig. 2a, sample 1). We ascribe this change to the increase of the refractive index of saline from 1.333 until 1.344 as salt reaches a concentration of 1 M^76^, and/or to a high density of surface-bound oligonucleotides. In the case of the 328-mer probe (Fig. 2a, sample 3), there occurred some particle aggregation, probably due to the higher concentration of NaCl that was needed to overcome the stronger repulsion (1.4 M). In the absence of any thiolated sequence, the citrate-AuNRs underwent massive and irreversible flocculation while dosing NaCl (Fig. 2a, sample 4).

**Table 1:** DNA sequences used as molecular probes and targets for the dot-blot assays and as PCR primers for *rol*C gene amplification.

Name	Sequence 5′ > 3′	Use
41-mer oligonucleotide probe	Thiol-(CH_2_)_6_-TTT TTT TTT TTT TTT TTT TTA TGG CTG AAG ACG ACC TGT GT	AuNR-probe, PCR primer
328-mer single-stranded probe	Thiol-(CH_2_)_6_-TTT TTT TTT TTT TTT TTT TTA TGG CTG AAG ACG ACC TGT GTT CTC TCT TTT TCA AGC TCA AAG TGG AGG ATG TGA CAA GCA GCG ATG AGC TAG CTA GAC ACA TGA AGA ACG CCT CAA ATG AGC GTA AAC CCT TGA TCG AGC CGG GTG AGA ATC AAT CGA TGG ATA TTG ACG AAG AAG GAG GGT CGG TGG GCC ACG GGC TGC TGT ACC TCT ACG TCG ACT GCC CGA CGA TGA TGC TCT GCT TCT ATG GAG GGT CCT TGC CTT ACA ATT GGA TGC AAG GCG CAC TCC TCA CCA ACC TTC CCC CGT ACC AGC ATG ATG TGA CTC TCG ATG A	AuNR-probe
*rol*C fw primer	ATG GCT GAA GAC GAC CTG TGT	PCR primer, target
*rol*C rev primer	TCA TCG AGA GTC ACA TCA TGC	PCR primer
DIG-labeled *rol*C fw primer	ATG GCT GAA GAC GAC CTG TGT-ddUTP-DIG	DIG-probe
*rol*C fw complementary sequence	ACA CAG GTC GTC TTC AGC CAT	Target
Mutated *rol*C fw complementary sequence_1	ACA CAG GTC GTC TTC AG–CAT	Target
Mutated *rol*C fw complementary sequence_2	ACA CAG GTC **T**TC TTC AG–CAT	Target
Mutated *rol*C fw complementary sequence_3	ACA CAG GTC **T**TC TTC **C**G–CAT	Target

All mutated complementary sequences include one deletion (–) plus none, one (G > **T**) or two (G > **T**; A > **C**) base substitutions.

The mean loading capacity of the 41-mer or the 328-mer probes on 0.8 mM Au AuNRs was quantified by spectrophotometry, and it was around 500 nM (corresponding to about 200 macromolecules per particle) and 16 ng/µl (i.e., about 80 nM, or 30 macromolecules per particle, which is in line with the typical rate of immobilizable immunoglobulins^9^), respectively. Even if no significant difference was observed in terms of ζ potential and size, the AuNRs functionalized with the 41-mer oligonucleotide displayed even greater electrophoretic mobility in TBE buffer than the control particles (Fig. 1b, sample 3). In fact, these particles migrated by about 2 cm towards the anode, with respect to 1.5 cm for the bare citrate-AuNRs, when exposed to 100 V for 60 min in 1% agarose gel, and their band was much narrower. Instead, when scanned by DLS, the AuNRs labeled with the 328-mer probe returned a hydrodynamic size around (157.4 ± 5.1) nm and signs of microscopic aggregation. The onset of flocculation in this system may be the main reason why it remained in its well during gel electrophoresis (Fig. 1b, sample 4).

Both the shorter and the longer sequences also stabilized the citrate-AuNRs in saline buffers or at high temperatures (Fig. 2b–e). The control sample visibly changed in color within few min after resuspension in DIG Easy Hyb buffer (b), which is the commercial solution implemented for standard detection of genetic targets with digoxigenin (DIG-11-ddUTP), and completely flocculated after 24 h (c) at room temperature. Aggregation usually started after 3 h in Dulbecco’s Phosphate-Buffered Saline (DPBS) in the absence of nucleotide probes (d). Bare particles also showed instability after 25 thermal cycles of a standard PCR run (e). In contrast, the bioconjugated AuNRs successfully endured all these quality tests.

### Dot-blot analysis

With the aim to establish a new rapid colorimetric sensor based on citrate-AuNRs functionalized with single-stranded DNA sequences of different length, a dot-blot assay was performed and compared against the method based on digoxigenin, which is the gold standard for labeling and detection. Digoxigenin is a steroid hapten that is in common use to label DNA probes for hybridization to membrane-blotted nucleic acids, according to standard methods^77^. The molecular hybrids are then immunodetected with anti-digoxigenin antibodies coupled to alkaline phosphatase (AP), and revealed with the colorimetric substrates NBT/BCIP (4-nitroblue tetrazolium chloride/5-bromo-4-chloro-3-indolyl phosphate).

We started with a model target designed as the 21-mer strand complementary to the PCR primer embedded in the first probe. First, the hybridization stringency was assessed by immobilizing decreasing concentrations (from 10 µM to 0.1 pM) of this target or point-mutated variants thereof. The same PCR primer was used as negative control in each membrane. All samples were prepared in duplicate to validate the reproducibility of the signals. As shown in Fig. S1 in Supplementary Information, colored dots appeared only after hybridization between the DIG-labeled oligonucleotide probe and its fully complementary sequence (line B) at 0.1 (B3, B4) and 1 nM (B1, B2). A single nucleotide deletion in this target was enough to prevent the development of any visible signal (line C), just as the further addition of one (line D) and two (line E) substitutions, even at the highest concentrations. No color emerged in the case of plain water, which rules out the effect of contaminations (column n.11).

**Figure 3:**
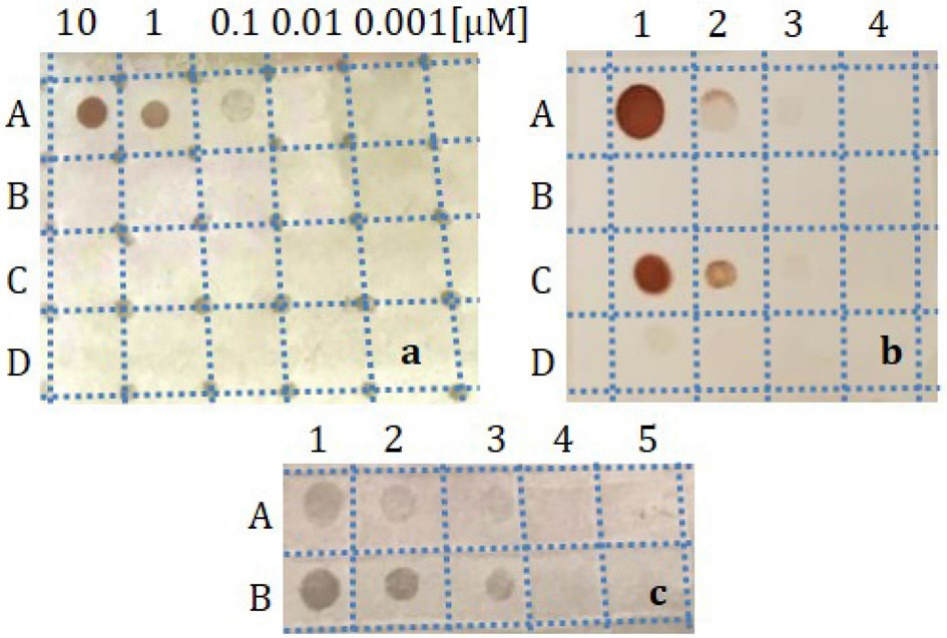
**(a)** Evaluation of the hybridization stringency between the 41-mer oligonucleotide bound to the AuNRs and its complementary sequence (A) spotted onto a nylon membrane. Targets with just two (B) or three (C) point mutations or completely identical to the oligonucleotide probe (D) did not allow any coloration to develop. **(b)** Colorimetric detection of the sequence complementary to the PCR primer bound to the AuNRs and used as positive control (A: 10, 1, 0.1, 0 µM), the same PCR primer chosen as negative control (B: 10, 1, 0.1, 0 µM), the *rol*C amplicon (C: 100, 10, 1, 0 ng/µl) and the pUC19: *rol*C plasmid (D: 50, 10, 1, 0 ng/µl) by means of AuNRs functionalized with the 41-mer oligonucleotide. **(c)** Dot-blot assay of plasmid DNA containing the *rol*C gene (A: 200, 100, 10, 1, 0 ng/µl) and the *rol*C amplicon (B: 100, 10, 1, 0 ng/µl) by implementing AuNRs functionalized with the 328-mer probe. The original and integral picture of the membrane is reported in Fig. S4B.

A high specificity of hybridization was also observed when labeling was made with AuNRs. Dots clearly appeared for concentrations exceeding 10 nM of the sequence fully complementary to the conjugated PCR primer (Fig. 3a, line A), and no signal arose from the mutated targets (Fig. 3a, lines B and C) nor from the negative control (Fig. 3a, line D).

In addition, the DIG-labeled probe enabled the recognition of a pUC19 plasmid containing the *rol*C transgene at 1, 10 and 50 ng/µl (Fig. S1 in Supplementary Information, F1–F6). Since the *rol*C sequence amounts to approximately 7% of the entire cloning vector, the minimum detectable concentration was about 70 pg/µl. Moreover, the intensity of the spots increased with the concentration of the target.

AuNRs conjugated to the 41-mer oligonucleotide allowed to easily identify a *rol*C amplicon until 1 ng/µl (Fig. 3b, line C). However, it took as much as 50 ng/µl of pUC19::*rol*C plasmid to raise a signal with this tag (Fig. 3b, D1). Also in this membrane, different concentrations of the same PCR primer bound to the AuNRs and its complementary sequence were respectively used as negative (line B) and positive (line A) controls.

In an attempt to enhance the sensitivity of detection of the plasmid DNA, we tested the use of the AuNRs conjugated to the 328-mer single-stranded sequence. By doing so, the detection limit decreased by about five folds down to 10 ng/µl, which corresponds to 0.7 ng/µl of *rol*C transgene, in this kind of sample (Fig. 3c, A3). Equal concentrations of the *rol*C amplicon were much more clearly visible when the AuNRs where conjugated to a longer probe (Fig. 3c, line B).

Our results demonstrate that labeling with AuNRs allows the detection of small amounts of DNA targets. Despite a lower sensitivity with respect to the use of digoxigenin, AuNRs provide much better speed and ease of use. When using AuNRs, a visible coloration already emerges within about 10 min of incubation, and saturates after few hours. Furthermore, AuNRs drastically streamline the steps and costs of the analytical protocol. After hybridization and standard stringency washes, the use of digoxigenin entails the preparation and implementation of blocking, antibody and color-substrate solutions, thus exploding the complexity of the workflow. The blocking and antibody solutions require a minimum of 30 min each. Then, after a washing step, the membrane must be incubated with the detection buffer for several hours in the dark. In fact, the colorimetric reaction starts within several minutes and saturates after 16 h, for reliable readout. Furthermore, the blocking and color-substrate solutions must be prepared as fresh as possible each time, and the antibody one is usable only within 12 h when stored at 2–8 °C. On the other end, the use of AuNRs allows real-time monitoring of the process of hybridization by the naked eye, because the hybridized probe is directly bound to the colorimetric tag. No preparation of buffers nor particular solutions are needed, but only post-hybridization washes may be desirable for more accurate use. If carefully stored at 4 °C, the same suspension of bioconjugated AuNRs is reusable multiple times over several weeks, without visible loss of colloidal stability nor staining efficiency. As a whole, the approach based on AuNRs is much less expensive than that relying on digoxigenin, and may enable new applications, such as e.g. real-time monitoring of the process of hybridization under dynamic conditions.

### Quantitative system for dot-blot image analysis

Together with the development of the plasmonic material, we tested the feasibility of a supervised machine learning paradigm to obtain quantitative predictions of the concentration of the target analyte from a photograph of a dot-blot assay.

**Figure 4:**
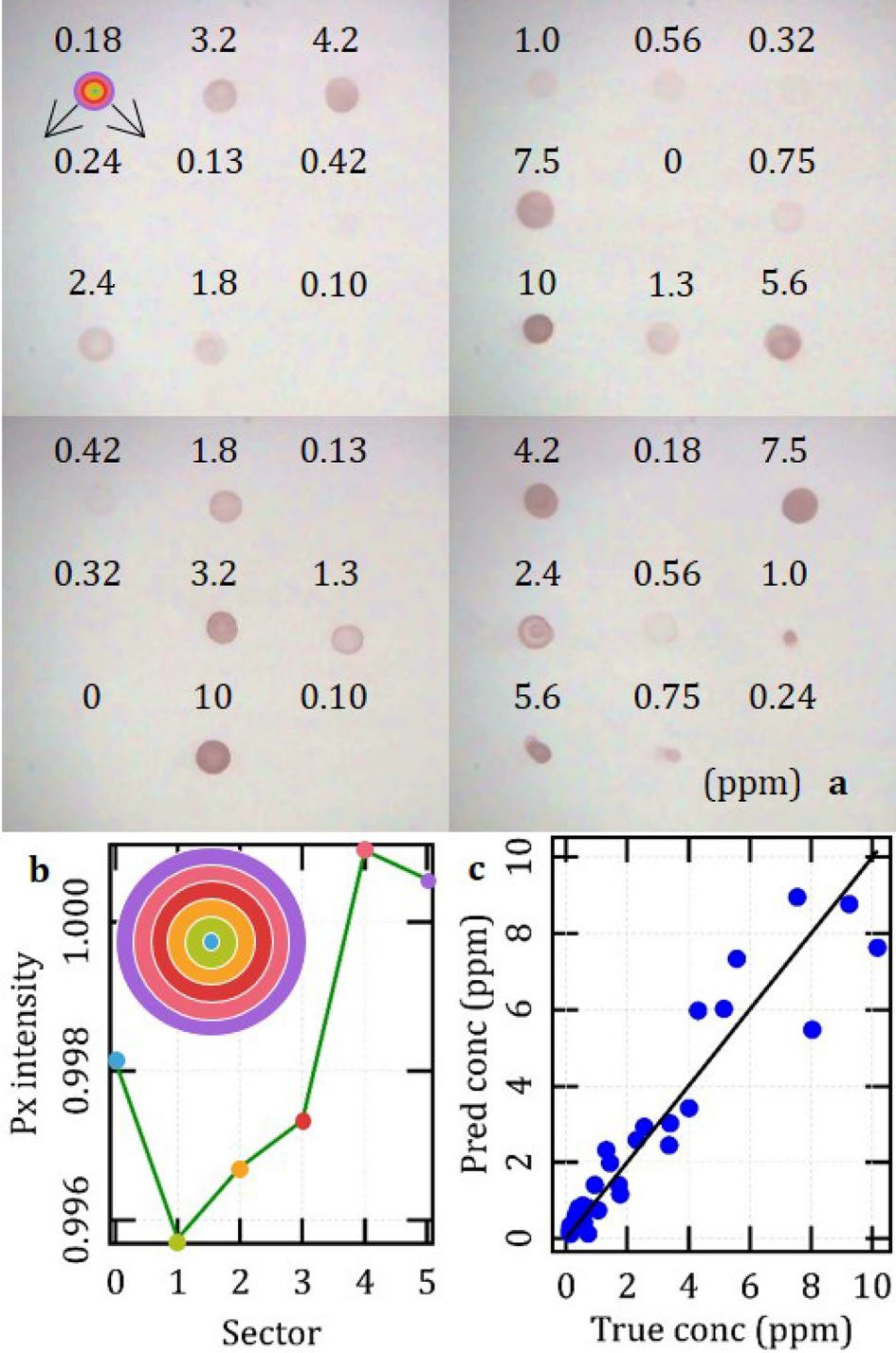
**(a)** standardized photographs of the entire data set of dot-blots. The multi-colored circle in the upper left corner is a mask used for feature selection, in full scale; **(b)** close-up of the mask highlighting its concentric layout (inset), and an example of an instance preprocessed with the mask. This case is a dot-blot corresponding to a target concentration of 0.24 ppm, which is invisible to the naked eye, but unveils a significant pattern upon feature selection; **(c)** results achieved with a cross-validation regressor based on a simple linear model, and expressed in terms of predicted vs true target concentration.

Figure 4 represents the preliminary pipeline that we fulfilled to automate the analysis of our dot-blots. First, we implemented a dedicated system to standardize the entire protocol from spotting to imaging of the membranes, and we captured a total of 36 pictures of dot-blots associated to different dilutions of the *rol*C amplified fragment by the PCR primer bound to the AuNRs, i.e. from 10 to 0.1 ppm and 0. According to the photographs shown in Panel 4a, dots come in irregular combinations of size and shape, sometimes featuring multiple coffee rings^78^, but altogether exhibit some pattern to the concentration of the target analyte. In particular, there appears to be a consistent trend for instances corresponding to densities exceeding about 1.0 ppm. Instead, nothing is clearly visible to the naked eye below 0.42 ppm. Figure 4a,b illustrate our procedure for feature selection based on averaging the pixel intensity over 6 consecutive rings through a digital mask, and so encoding each dot-blot as a low-dimensional vector representing its spot brightness from center to periphery. As an example, Fig. 4b refers to the bottom-rightmost instance in Fig. 4a and suggests that this representation may help expose subtle patterns that may elude a qualitative inspection. Fig. 4c summarizes the results achieved by applying a cross-validation approach based on a simple linear model to the regression of our dataset. In particular, each data point was associated to a clean prediction derived from a so-called leave-one-out regressor trained with all other 35 instances. As a downside, this also means that the graph displayed in panel 4c actually integrates results obtained from 36 different models, i.e. it is not a full regressor.

The cross-validation predictions feature a mean absolute error (MAE) with respect to the true labels of 0.59 ppm. However, it turns out that the discrepancy tends to decrease with concentration, in absolute terms (see Fig. S2 in Supplementary Information). For instance, over the range from 0 to 1 ppm, it amounts to a MAE of 0.24 ppm, which we take as an indicator of the detection limit and sensitivity of our demonstrator. In relative terms, we note that metrics tend to improve with concentration, though. Above 2 ppm, the relative error tends to converge to an average value around 19% (see Fig. S2).

Our results collectively prove the feasibility of an approach based on a supervised machine learning pipeline to obtain a quantitative readout of our dot-blots. Meanwhile, with respect to an assay based on a visual assessment, it holds the potential to tweak its detection limit by about twofold, say from around 0.42 to 0.24 ppm. We also note that the average of the mean absolute train errors over the 36 leave-one-out models amounts to 0.40 ppm, which is about 30% lower than their mean absolute test errors, as reported above, thus pointing to a systematic tendency to overfit their respective input datasets. Therefore, since the linear assumption is already likely to oversimplify the physical process, thus bearing some bias, we expect that a larger dataset will allow much better predictions, and will make it reasonable to try hyperparametric models. Another improvement may come from a refinement of the system hardware and all methods for standardized imaging. Panel 4a clearly shows that there remains further scope to optimize the alignment of the dot-blots and the illumination of the substrate, in order to gain consistency. Overall, we are confident that a sensitivity below 100 ppb will probably be targetable without too much effort.

## Methods

### Materials

Cetrimonium bromide, tri-sodium citrate dihydrate, poly(sodium 4-styrenesulfonate), Tween 20, Tris(2-carboxyethyl)phosphine hydrochloride, sodium chloride, sodium dodecyl sulfate, agarose, Tris–Borate EDTA buffer, and all chemicals for the synthesis of AuNRs were purchased from MilliporeSigma (Merck KGaA, Germany). Dulbecco’s Phosphate-Buffered Saline was supplied by PAN-Biotech (Germany). Unmodified PCR primers and all complementary target sequences were obtained from Eurofins Genomics (Eurofins Scientific, Germany) and dNTPs from BioChain (BioChain Institute, USA).

### Preparation of gold nanorods

Cetrimonium-coated AuNRs were synthesized according to the prescriptions reported by X. Ye et al.^79^, and in particular the case featuring 47 mM CTAB, 8.3 mM sodium oleate, 180 µM AgNO_3_, 3.4 mM HCl and 0.038% seed solution, which conveys an average AR around 3.9.

In order to obtain an anionic electrokinetic potential, CTAB was gradually replaced with Na_3_-citrate, by following and modifying the procedure reported by Mehtala et al.^56^. In our modification, AuNRs were initially incubated with an aqueous solution of 0.15% Na-PSS, also containing 0.5 mM CTAB, which confers more colloidal stability in this critical passage, for 24 h at RT under gentle stirring. We speculate that in this step other polyanions may be implemented instead of Na-PSS, such as poly(acrylic acid)^80^. After three cycles of centrifugation at 11.000*×*g and resuspension in 0.15% Na-PSS, AuNRs were transferred into an aqueous solution of 5 mM Na_3_-citrate, left at rest for further 24 h, purified by two cycles of centrifugation, and finally stored at a nominal concentration of 1.6 mM Au in 5 mM Na_3_-citrate, until use.

Citrate-stabilized AuNRs were characterized by DLS (NANO-ZS90 Zetasizer, Malvern Instruments, UK), UV–Visible spectrophotometry (BioSpectrometer basic, Eppendorf, Germany) and gel electrophoresis (PerfectBlue Horizontal Mini Gel System, PEQLAB Biotechnologie, Germany).

### Preparation of DNA probes

The nucleotide probes chosen for bio-conjugation to the AuNRs are displayed in Table 1.

The first one consisted of a synthetic 21-mer DNA oligonucleotide, which was modified with a tail of 20 thymine bases and a terminal thiol at its 5′-end (Metabion International AG, Germany). This short sequence was also used as specific forward primer for *rol*C gene in an end-point PCR process (T100 Thermal Cycler, Bio-Rad Laboratories, USA).

The second probe was a 328-mer single-stranded DNA sequence, which was derived from a 328-bp double-stranded *rol*C amplified fragment. The template for PCR amplification was the pUC19 cloning vector (New England Biolabs, USA), containing a 1860-bp *Agrobacterium rhizogenes* (strain 1855) HindIII-EcoRI restriction fragment that encompassed both the promoter and the coding region of *rol*C gene (ORF12)^81^. The HindIII-EcoRI fragment was cloned into the pUC19 polylinker restriction sites and ligated with T4 DNA ligase (New England Biolabs, USA). About 1 ng of this template was amplified by end-point PCR with the specific thiol-labeled forward and an unmodified reverse primer at an annealing temperature of 55 °C, in several replicates. In this manner, amplicon molecules were generated with terminal thiols at the 5′-ends of their sense strands. A typical PCR reaction mixture contained 200 nM of each primer (Table 1), 800 µM dNTPs and 0.05 U/µl DreamTaq DNA Polymerase (Thermo Fisher Scientific, USA). At the end of 35 cycles, each product was purified by MicroSpin columns (Illustra GFX PCR DNA and gel band purification kit, GE Healthcare, UK), according to the instructions provided by the manufacturer and by eluting with 10 mM Tris–HCl buffer (pH 8.0). The concentration of the purified amplicon was finally quantified by a Qubit fluorometer (Invitrogen, Thermo Fisher Scientific, USA).

### Functionalization of gold nanorods with DNA probes

The stock suspension of citrate-stabilized AuNRs was transferred into a 0.5 mM Na_3_-citrate solution containing 0.005% Tween 20, at the nominal concentration of 0.8 mM Au.

In the meantime, both thiolated nucleotide probes were treated with 10 mM Tris(2-carboxyethyl)phosphine hydrochloride (TCEP) for 1 h at RT, in order to reduce potential disulfide bridges into more reactive thiols. A thermal denaturation step was required for the *rol*C amplicon to split into single strands.

About 4 µM thiolated primer or at least 40 ng/µl denatured amplicon were added to the suspension of AuNRs and incubated for two days at 4 °C under constant agitation. In an attempt to reduce the electrostatic repulsion between the anionic AuNRs and the phosphate moieties of the nucleotides, a gradual addition of NaCl was performed until 1–1.4 M, according to the length of the thiolated sequence. Before use, AuNRs were washed four times to ensure the complete removal of unbound probes. The amount of each probe immobilized onto the AuNR surface was quantified by spectrophotometry at a wavelength of 260 nm, with reference to known concentrations of the individual components. Finally, AuNRs were re-suspended in a hybridization buffer optimized for dot-blot assays at the nominal concentration of 0.8 mM Au.

### Preparation of DNA targets and dot-blot assays

In order to assess the biomolecular recognition between the AuNR-labeled probes and specific target samples, a dot-blot assay was performed and compared to a standard method for labeling with digoxigenin and detecting with an enzyme immunoassay^82^.

1 µl of each target was spotted onto positively charged nylon membranes and fixed for 3 min by an UV-crosslinker (Spectrolinker XL-1000 UV Crosslinker, Spectronics Corporation, USA). In order to establish the hybridization stringency, different oligonucleotides were immobilized in a range of concentration from 0.1 pM to 10 µM, i.e. sequences complementary to the 21-mer oligonucleotide probe or featuring one or more mutations (base substitutions and/or deletions, see Table 1). The *rol*C fw primer and plain water were used as negative hybridization and contamination controls, respectively. In addition, the PCR product (1, 10 and 100 ng/µl) and plasmid DNA containing the *rol*C gene (1, 10, 50, 100, 200 ng/µl) were also spotted after thermal denaturation at 95 °C for 10 min and rapid cooling in ice.

The DIG-labeled probe was prepared according to the instructions of the “DIG oligonucleotide 3′-end labeling” kit (2nd generation, Roche Diagnostics, Germany). A pre-hybridization of membranes was performed at 54 °C for 30 min with an appropriate volume (20 ml/100 cm^2^) of a ready-to-use buffer (DIG Easy Hyb, Roche Diagnostics, Germany) in a hybridization oven (Techne, UK). An overnight hybridization was then done at the same temperature with at least 3.5 ml/100 cm^2^ DIG Easy Hyb buffer containing 200 pmol DIG-labeled probe. The next day, membranes were rinsed twice with a 2× saline-sodium citrate (SSC) solution supplemented with 0.1% sodium dodecyl sulfate (SDS) for 5 min at RT, followed by two high stringency washes in 0.1 × SSC buffer with 0.1% SDS for 15 min at 65 °C under constant agitation. The immunological detection was finally achieved by using an anti-digoxigenin antibody conjugated to alkaline phosphatase, in the presence of the colorimetric substrates NBT/BCIP, as described in the procedure of the “DIG DNA labeling and detection” kit (Roche Diagnostics, Germany).

For colorimetric detection with the AuNRs, after pre-hybridization, membranes were incubated with relevant suspensions conjugated with the 41-mer or 328-mer sequences at the nominal concentration of 0.8 mM Au in DIG Easy Hyb buffer at 54 °C under gentle agitation, until development of a visible color. Then, samples were just directed to post hybridization washes, rinsed with distilled water and air-dried.

### Dot-blot setup for standardized measurements

In order to develop a reproducible system for quantitative analysis of the dot-blots based on an automated pipeline, additional membranes were prepared by using a system that was purposely designed and produced by Ecobioservices and Researches (Italy), in collaboration with Laboratori Victoria (Italy).

**Figure 5:**
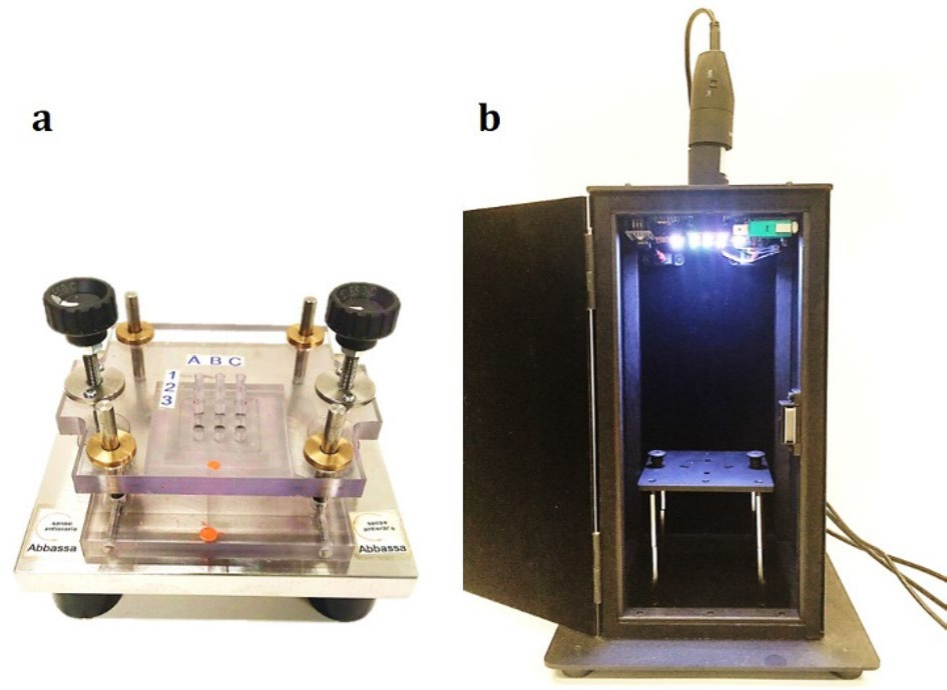
Photographs of the dispenser **(a)** and the darkroom **(b)** manufactured by Ecobioservices and Researches and Laboratori Victoria.

First, nylon membranes with identical shape and size (3.6 cm × 3.6 cm) were created by using a custom-made cutter (Fig. S3c in Supplementary Information). Then, after thermal denaturation, serial dilutions of the purified *rol*C amplicon (from 10 to 0.1 ppm with a dilution factor of 4/3, and 0) were spotted onto these membranes in duplicate and in random order. In order to guarantee a uniform and reproducible deposition of all target samples and to minimize the variability due to manual pipetting, we implemented an appropriate dispenser (Fig. 5a, Fig. S3a) made of a steel plate and a PMMA grid featuring 9 through-holes arranged in a 3 × 3 matrix. The steel plate includes a housing for the nylon membranes to settle in position. During dispensing, the PMMA grid was placed above the steel plate and its holes were used as a guide for a micropipette tip, in order to standardize the positions of the dots, and to streamline the subsequent analysis. A fine movement is available to control the distance between steel plate and PMMA grid. In this manner, each sample was set at a consistent pitch distance of its neighbors (about 0.9 cm).

As described above, membranes were UV-crosslinked, pre-hybridized and treated overnight with a suspension of AuNRs conjugated with the 41-mer oligonucleotide, at the nominal concentration of 0.4 mM Au. Low and high stringency washes were then carried out as usual. After air-drying, images of the dot-blots were captured using a digital camera mounted inside a darkroom designed to ensure a reproducible illumination and acquisition. The darkroom consists of a cabinet of a size of 15 cm × 15 cm base × 24.5 cm height and featuring a sealed door and a slot for correct insertion of the membranes (see Fig. 5b, Fig. S3b). For uniform illumination, an array of 12 LEDs was installed around the digital camera in the upper wall of the cabinet. The camera was placed at a distance around 23 cm of the membranes and connected to a PC via USB. A custom-made software allows the user to control the intensity and RGB composition of the LEDs, and to acquire and store standardized photographs.

### Analysis of dot-blot photographs by artificial intelligence

In order to check the feasibility to analyze the standardized photographs in the framework of a supervised machine-learning problem, we assessed the sensitivity of a pipeline that receives an image of a dot-blot membrane as input and returns a quantitative prediction of the concentration of the nucleotide target as output. The extreme limitation of the available dataset (n = 36 instances) imposed a strong reduction of its dimensionality, a preference for the simplest possible model, and a workflow circumventing the need of splitting into train and test. Our objective was to understand the relevance of machine learning in our system, rather than to deploy an up-and-running predictor.

For dimensionality reduction, image segmentation and feature selection, photographs were first resized to greyscale and then preprocessed with a tool that leverages on the theoretical symmetry of the physical problem (see Fig. 4b). It consists of a mask made of 6 concentric rings, each of a Chebishev thickness of 6 px. In terms of physical size on the substrate, this corresponds to an outer diameter of 3.2 mm and distance from ring to ring of 270 µm, which approximately suit the max width of the dots and the estimated lateral res of the imaging system. For each ring, the avg px intensity was computed, normalized to the value found at the outer edge of the mask and plotted vs numeral distance from the common center, i.e. from 0 to 5. These plots were also used to align the mask by fine-tuning its center around a nominal position, in a way to maximize their variance or, in other words, to minimize their blurriness. The assumption that this criterion optimizes the overlap with the physical pattern was checked by manual inspection. Therefore, each instance was reduced from a matrix of an approximate size of (70 × 70) px × 3 colors ~ 15 × 10^3^ values to a vector of length n = 6 dimensions. Finally, in an attempt to further mitigate the effect of sample variance, each label was modified with a random tweak (5%) within the physical uncertainty of the pipette (at least 10%).

In order to deploy a supervised machine learning analysis, we used Scikit-learn^83^ as a high-level environment comprising tools for performance assessment. In particular, we tested the simplest model of linear regressor and calculated its performance metrics with a cross-validation analysis implemented in a leave-one-out strategy, i.e. an extreme case of K-fold cross validation done with K = n. For each K-fold iteration, Scikit-learn provides both a test and a train error, which we compared for an overall assessment of its generalization error. We also used the cross-validation tool to generate so-called clean predictions, where the concentration of each instance was inferred from a sub-model trained with all other 35 cases. We examined these predictions to identify relevant trends vs true concentration, and to gain an indicative estimate of the sensitivity of our simple predictor. However, we declined to deploy and test a full regressor, because of too small a size of the available dataset.

### Consent for publication

Written informed consent for publication was obtained from all participants.

## Conclusions

In conclusion, we have conjugated citrate-terminated gold nanorods with two different nucleotide probes and implemented a setup that pursues a rapid, accurate and reproducible method of detection of a genetic target. Although the use of gold nanorods implies a lower sensitivity than the gold-standard method based on digoxigenin, our system may be enough for use in critical contexts like the diagnosis of most infectious diseases, where the pathogen concentration is relatively high. It works fine with a model target, as well as PCR products and plasmid DNA, and it is stringent against single point mutations. The main advantage is a much more streamlined procedure, which may also open new possibilities, like the real-time observation of biorecognition events occurring under dynamic conditions.

We have also discussed the perspective to analyze the dot-blot membranes with a supervised machine learning approach implemented after a dedicated methodology for the acquisition of standardized photographs. A simple linear regressor built over a dataset of as few as 36 dots already returned leave-one-out cross-validation predictions with a MAE of 0.59 ppm, which decreases to 0.24 ppm in the range of concentration of the target analyte below 1 ppm. These results are rather surprising, when it is considered that the starting point was a naked-eye detection limit around 0.42 ppm, and that the entire pipeline from system hardware for standardized imaging to the accumulation of an adequate training dataset and the optimization of the regression model leaves substantial room for improvement.

In conclusion, our work demonstrates the feasibility of a collective revision of the paper-based format built upon a synergistic combination of complementary enhancements: (1) The use of gold nanorods or other anisotropic shapes, which give intrinsic 10× brightness with respect to standard spherical particles, and are multiplexable. (2) The optimization of a genetic strategy, where we made a 5× improvement of the detection limit of a transgene cloned into a plasmid by just tuning the chain length of a synthetic probe. And (3) the implementation of a pipeline for quantitative readout based on machine learning, where an early test already returned another 2× upgrade of the sensitivity reached by the naked eye. Taken together, we are confident that our work will motivate more efforts to integrate recent advances in nanobiotechnology and data processing into topical tools like the so-called rapid antigen-tests for SARS-CoV-2, in order to boost their analytical performance and draw closer to the level of lab-grade methods.

## Supplementary Information

Supplementary Figures.

## Data Availability

Data are available at the following link: https://cloudbnlab.ifac.cnr.it/s/6rTd7SP46kgJ9y7.
