# Supplementary figures and images for: Paper-based genetic assays with bioconjugated gold nanorods and an automated readout pipeline
Source: Sci Rep. 2022 Apr 13;12:6223. doi: 10.1038/s41598-022-10227-7 (PMC9007582; doi:10.1038/s41598-022-10227-7)

Supplementary Information

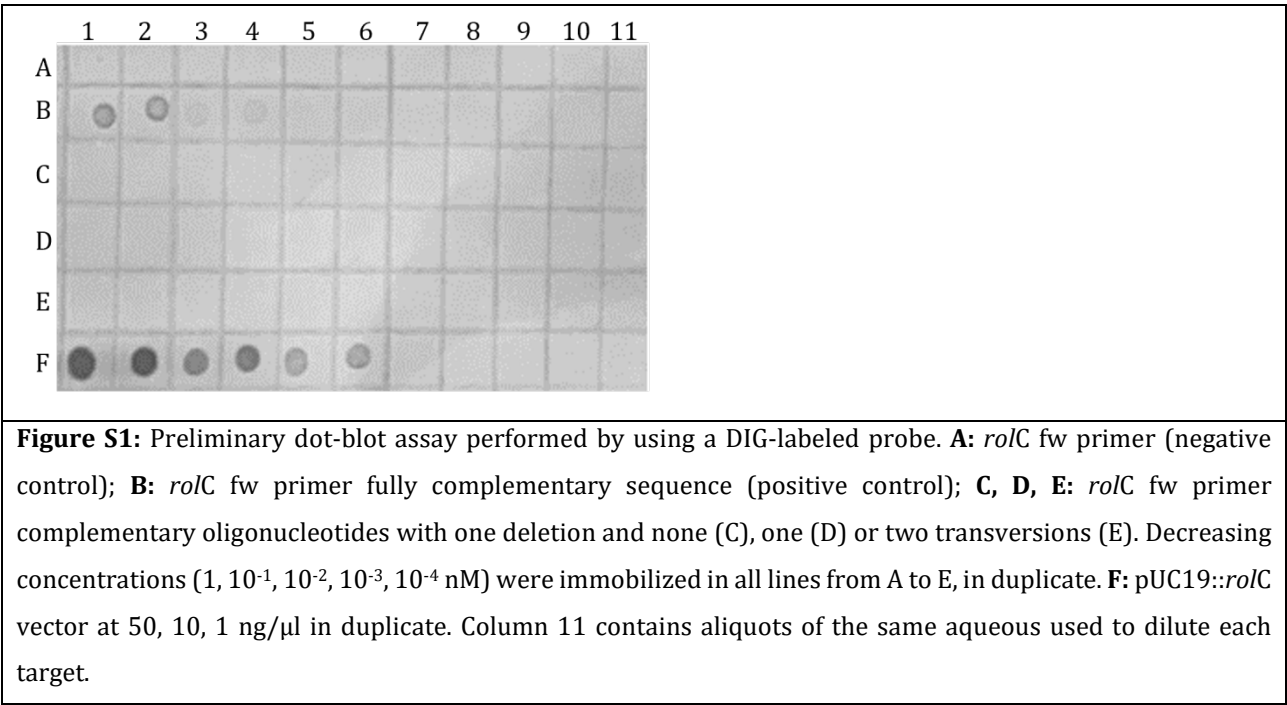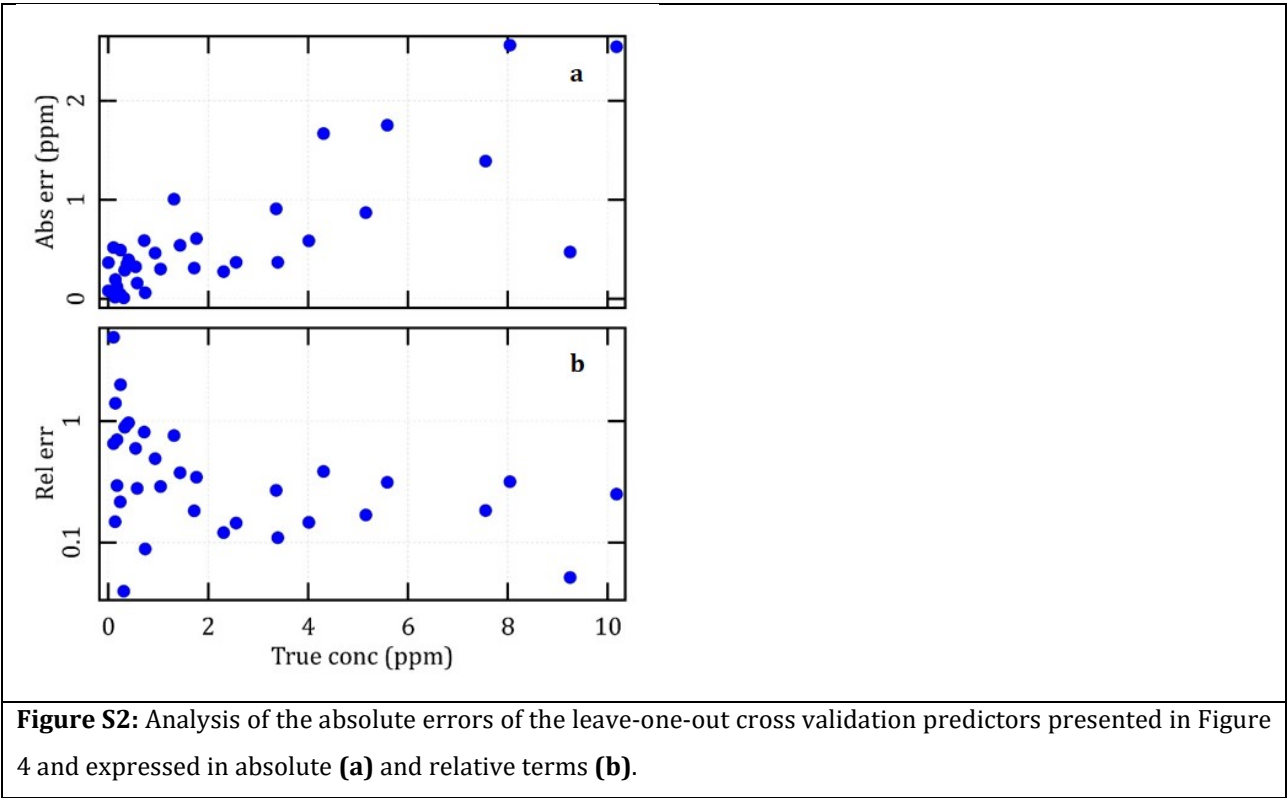

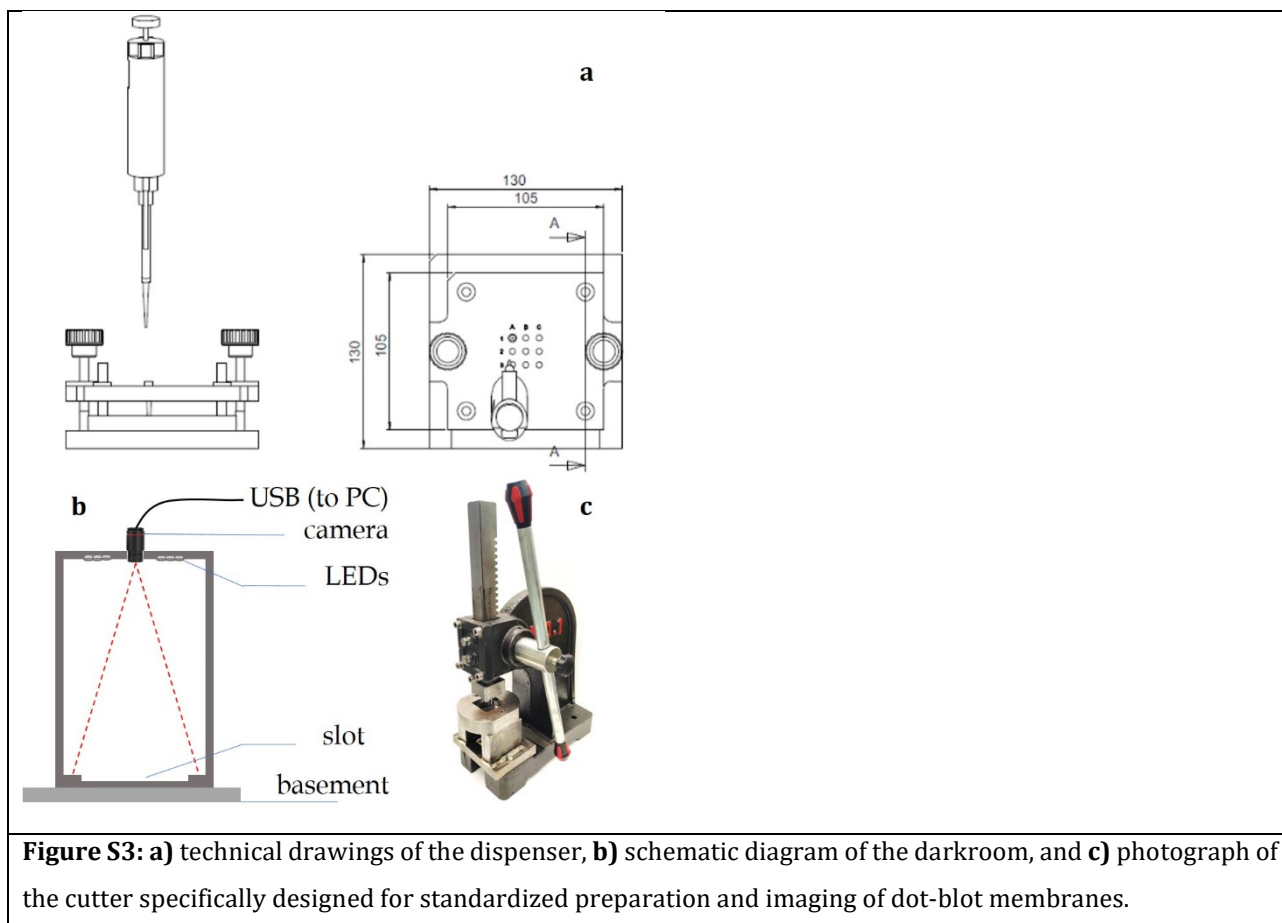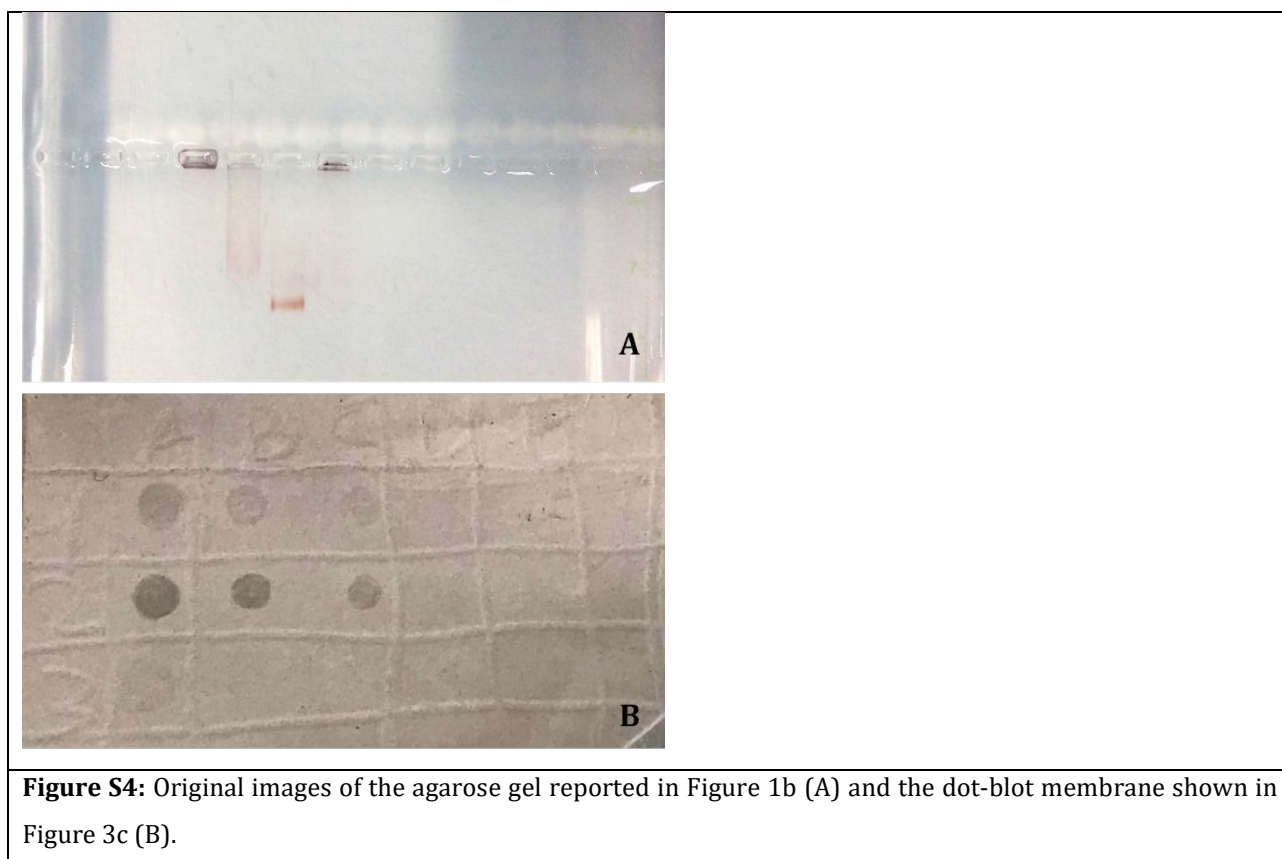

Supplement: Supplementary file 1 — Supplementary Figures. [file 41598_2022_10227_MOESM1_ESM.pdf]
